# Identification and Characterization of Immune-Associated MicroRNAs in Silver Carp (*Hypophthalmichthys molitrix*) Responding to *Aeromonas veronii* and LPS Stimulation

**DOI:** 10.3390/ani14020285

**Published:** 2024-01-17

**Authors:** Meng Liu, Huan Tang, Kun Gao, Xiqing Zhang, Zihan Yang, Yunhang Gao, Xiaofeng Shan

**Affiliations:** College of Veterinary Medicine, Jilin Agricultural University, Changchun 130118, China; liumeng4610@163.com (M.L.); tanghuan202308@163.com (H.T.); kungao213@163.com (K.G.); zhangxiqing1020@163.com (X.Z.); yyangzihan622@163.com (Z.Y.)

**Keywords:** microRNA, *Aeromonas veronii*, LPS, silver carp, immune response

## Abstract

**Simple Summary:**

Infections caused by pathogenic bacteria are one of the main problems in silver carp (*Hypophthalmichthys molitrix*) aquaculture, resulting in elevated mortality rates and substantial economic losses. Therefore, it is of great significance to investigate the response of silver carp to pathogenic bacterial infection and its underlying regulatory mechanisms. MicroRNAs (miRNAs) play an important role in regulating the immune response of teleost fish. In this study, we established an inflammatory model in silver carp and employed bioinformatics analysis to identify miRNAs with immunomodulatory effects. The data obtained from this study lay a solid foundation for elucidating the regulatory function of silver carp miRNAs during bacterial infection, while also providing valuable insights into the molecular mechanisms governing miRNA adaptation in teleost fish during pathogen invasion.

**Abstract:**

The ubiquitous Gram-negative bacterial pathogen *Aeromonas veronii* (*A. veronii*) can easily cause inflammatory reactions in aquatic organisms, resulting in high mortality and huge economic losses. MicroRNAs (miRNAs) participate in immune regulation and have certain conserved properties. MiRNAs are involved in the immune responses of a variety of teleost fish infected with bacteria, whereas there is no related report in silver carp (*Hypophthalmichthys molitrix*). Therefore, we identified the expression profiles of miRNA in silver carp stimulated by *A. veronii* and LPS. Among them, the quantity of differentially expressed miRNAs (DEmiRNAs) obtained in the silver carp challenge group was 73 (*A. veronii*) and 90 (LPS). The GO enrichment and analysis of KEGG pathways have shown that the predicted target genes are mainly associated with lipid metabolism and the immune response in silver carp. This indicates the possibility that miRNAs play a role in regulating immune-related pathways. In addition, a total of eight DEmiRNAs validated the accuracy of the sequencing result via quantitative real-time PCR (qRT-PCR). Finally, we selected the silver carp head kidney macrophage cells (HKCs) as model cells and proved that miR-30b-5p can regulate the inflammatory response in silver carp HKCs. This study lays the foundation for exploring miRNA regulation in silver carp during pathogenic bacterial infection. In addition, it provides a reference for the future development of non-coding RNA antibacterial drugs.

## 1. Introduction

Silver carp (*Hypophthalmichthys molitrix*) is one of the most significant freshwater aquaculture fish distributed across all major Chinese water systems [1]. Moreover, silver carp is an inexpensive species, being especially preferred by aquaculture farmers. As a typical filter feeder, silver carp feeds on aquatic plankton and inhibits the growth of blue-green algae, which also plays a positive role in improving water quality [2]. However, with the rapid growth of industrial aquaculture, silver carp culture has become susceptible to a variety of diseases caused by bacteria, parasites, and viruses [3]. *Aeromonas veronii* (*A. veronii*) is a ubiquitous Gram-negative bacterium in nature, especially in aquatic environments, which can cause hemorrhagic septicemia and ulcerative conditions in many aquatic and terrestrial animals, including humans [4]. *A. veronii* can infect many fish species, and as a conditional pathogen, *A. veronii* is more likely to cause infections in aquatic organisms with compromised immunity or traumatic body surfaces, which can lead to high mortality and huge economic losses [5,6,7]. Lipopolysaccharide (LPS), the main component of the outer membrane of Gram-negative bacteria, is mainly used to induce inflammation models both in vitro and in vivo [8]. However, little is known about the role of innate immunity in the antibacterial responses of silver carp under LPS stress. Hence, an understanding of the immune system and pathogen defense mechanisms in silver carp is essential for the establishment of effective disease control measures to reduce financial losses.

MicroRNAs (miRNAs) are endogenous small non-coding RNAs spanning around 18–23 nucleotides. As a subject of in-depth study, they have expanded to include a wide range of organisms since the first miRNA was found in *Caenorhabditis elegans* in 1993, encompassing mammals [9], plants [10], viruses [11], teleosts [12], etc. Most of the primary miRNAs are produced by the transcription of DNA sequences and produce precursor miRNAs after processing, which finally convert into mature miRNAs. MiRNAs are important post-transcriptional regulators of gene expression, which can interact with the 3′ untranslated region (3′UTR) of target mRNAs to arouse mRNA degradation and impede translation [13]. In addition, research has shown that miRNAs can also interact with other regions, and, in some cases, miRNAs can also activate translation [14]. Increasing amounts of data point to the importance of miRNA-mediated control.

With deeper research on miRNA, new techniques and methods have been developed and applied, including high-throughput sequencing technology, which is widely used as an economical, efficient, and sensitive experimental method [15]. At present, this technology has been widely used in the identification and detection of miRNA in many types of fish. Huang et al. used deep sequencing and discovered 324 miRNAs in the kidneys, spleen, muscle, and liver of the blunt snout bream (*Megalobrama amblycephala*), comprising 15 novel miRNAs and 309 conserved miRNAs [16]. High-throughput sequencing technology was used in largemouth bass (*Micropterus salmoides*) to comprehensively analyze the miRNA–mRNA, and 13 differently expressed miRNAs related to lipid and glucose metabolism were identified in the liver under conditions of acute hypoxia [17]. Increasing evidence suggests that miRNAs are widely present in organisms and participate in a series of key biological processes, including inflammation, the stress response, and immunity [18,19]. Previous studies have identified the miRNA expression profiles during bacterial infection in teleosts, such as turbot (*Scophthalmus maximus* L.) infected with *Vibrio anguillarum* [20], yellow catfish (*Pelteobagrus fulvidraco*) infected with *A. veronii* [21], and zebrafish (*Danio rerio*) infected with *Streptococcus parauberis* [22]. Apart from this, miRNA can also modulate the production of inflammatory cytokines through signal transduction, thereby regulating the antibacterial immune response. For instance, microRNA-182-3p negatively regulates the generation of inflammatory factors by targeting TLR5M, consequently participating in the inflammatory response [23]. Collectively, previous studies have shown that miRNAs in teleost fish play an important role in regulating bacteria-induced signaling. It is notable that the role of miRNA in silver carp pathogenic bacterial infection has not yet been discussed. Furthermore, the regulatory mechanisms of miRNAs in the immune response pathways of teleost fish also require further exploration.

In this study, a combined analysis of Gram-negative bacteria (*A. veronii*) and its single-component LPS infection model in silver carp is reported for the first time. Differentially expressed miRNAs (DEmiRNAs) with the same regulatory trend were screened, and it was speculated that they may have a broad-spectrum regulatory effect on Gram-negative bacterial infection. GO enrichment and KEGG pathway analysis have shown that the predicted target genes are associated with the pathways implicated in lipid metabolism and immunity in silver carp. The obtained data lay the foundation that aids us in elucidating the regulatory role of silver carp miRNA in Gram-negative bacterial infection. Meanwhile, it provides a reference to further study the molecular mechanism behind teleost fish miRNA adaptation during pathogen infection.

## 2. Materials and Methods

### 2.1. Ethical Approval

The experimental animal standard schemes used in this study follow the JLAU Animal Experiment Regulations (JLAU08201409). Every step of this study’s procedure follows the ARRIVE guidelines https://arriveguidelines.org (accessed on 2 March 2022) and the National Research Council’s Guide for the Care and Use of Laboratory Animals.

### 2.2. Experimental Fish and Sampling

Healthy silver carp (52.23 ± 3.09 g) were purchased from Jiutai Experimental Farm, Jilin Academy of Fisheries Sciences. Before the experiment, silver carp were domesticated for 2 weeks under freshwater conditions in a laboratory (water temperature: 25 ± 2 °C; dissolved oxygen: over 4 mg/L, pH 7.0–7.5). The water quality was maintained via mechanical filtration, and the freshwater was flow-through aerated. Fasting was performed for 3 days before the infection experiment, and the spleens of silver carp were randomly extracted for bacterial isolation to ensure no other pathogenic bacteria infection.

*A. veronii* was a laboratory preservation strain (AMQ40920.1). Referring to the previous description [24], the infection concentration was determined by the median lethal dose (LD50) of bacterial infection, and the bacterial suspension was prepared using sterile physiological saline. Three groups of silver carp were created at random: *A. veronii* group (AV), LPS group (LPS), and control check group (CK). Each group consisted of 10 silver carp (6 as experimental, and the rest for supplementation). Silver carp were injected intraperitoneally with 200 μL of *A. veronii* (3.7 × 10^6^ CFU/mL) and LPS (1 mg/mL, InvivoGen, San Diego, CA, USA). Injections of sterile saline at the same dose were administered intraperitoneally to silver carp in CK, and the spleen tissues from silver carp in each group were immediately collected 24 h after injection. The silver carp were anesthetized with MS-222 (100 mg/L, Sigma-Aldrich, St. Louis, MO, USA) [25] before the tissues were collected. The obtained tissues were immediately held in liquid nitrogen for more than 15 min and kept at −80 °C for RNA extraction and sequencing.

### 2.3. Total RNA Extraction, miRNA Library Construction, and Sequencing

Total RNA was isolated from each group from spleen samples at a 24 h time point using the Trizol kit (Invitrogen, Carlsbad, CA, USA), as directed by the manufacturer. Genomic DNA contamination was removed using RNase-free DNase I (TaKaRa, Osaka, Japan). RNA integrity was evaluated via agarose gel electrophoresis. The quality and concentration of the RNA were measured using a spectrophotometer (Nano Drop Technologies, Wilmington, DE, USA), and the completeness of the RNA was assessed by employing a bioanalyzer (Agilent Bioanalyzer 2100, Pleasanton, CA, USA). Then, the TruSeq Small RNA Sample Prep Kits (Illumina, San Diego, CA, USA) were used to prepare the miRNA sequencing library. Finally, the constructed library was sequenced using Illumina Hiseq 2000/2500 (Illumina, San Diego, CA, USA), and a single-ended reading code of 50 bp was sequenced.

### 2.4. Identification and Analysis of miRNAs

Raw reads of the miRNA were examined using the software ACGT101-miR (v4.2, LC Sciences, Houston, TX, USA) to remove the sequences with splice contamination, unidentifiability, and a base ratio greater than 10% in the raw data. Among the remaining sequences, those with 18–32 nucleotides were retained. Thereafter, a variety of RNA databases, including the mRNA database, Repbase database https://www.girinst.org/ (accessed on 26 May 2022), and RFam database (v14.7) https://rfam.xfam.org (accessed on 26 May 2022), were used to compare and filter the remaining sequences (apart from miRNA). The final valid data were used for a subsequent analysis of the miRNA. The effective sequence was mapped to the reference full-length transcriptome of silver carp, and miRBase (v22.0) https://mirbase.org/ (accessed on 26 May 2022), was used to obtain known miRNAs. For secondary structure prediction, the mfold program was utilized to obtain novel miRNAs. Finally, the Ensembl and miRBase databases were used to annotate the sequences of known and novel predicted miRNAs.

### 2.5. Screening and Analysis of DEmiRNAs

The identified known and novel predicted miRNA sequences were counted for TPM (transcript per million sequences); i.e., the TPM were normalized to obtain the normalized value [26]. Since the measured samples had biological repetition, a method based on normal distribution was used to calculate the *p*-value. The *t*-test was carried out to compare the DEmiRNAs between the infected and CK groups, and *p* < 0.05 was used as the threshold. With log2 (FC) as abscissa and log10 (*p*-value) as ordinate, the volcano map was drawn for all miRNAs in differential expression analysis.

### 2.6. Prediction and Analysis of miRNA Target Genes

Two miRNA target prediction databases, i.e., TargetScan (v5.0) [27] and miRanda (v3.3a) [28], were utilized to predict the targets of DEmiRNAs. Ultimately, the target gene of DEmiRNAs was determined to be the intersection of these two pieces of software.

Kyoto Encyclopedia of Genes and Genomes (KEGG) and Gene Ontology (GO) were used to enrich the functional annotations of miRNA target genes in order to pinpoint the main signal transduction pathways and biochemicals involved. The OmicStudio tools https://www.omicstudio.cn/tool/56 (accessed on 27 June 2022) were used to create the miRNA–mRNA network graphic.

### 2.7. Verification of miRNA Expression through Quantitative Real-Time PCR (qRT-PCR)

A total of 8 DEmiRNAs were chosen for verification with the aim of verifying the outcomes of high-throughput sequencing. The primer information for quantitative real-time PCR (qRT-PCR) is shown in Table S1. Total RNA extraction was carried out using spleen samples used to construct miRNA libraries. MiRNA was isolated from total RNA using an RNAiso for Small RNA Kit (Takara, Beijing, China) and reverse-transcribed into first-strand cDNA using the Mir-X™ (Vazyme, Nanjing, China). Then, the fluorescence quantification of reverse-transcribed cDNA was performed in an ABI 7500 real-time PCR system (ABI, Los Angeles, CA, USA) using TaKaRa Taq HS Perfect Mix reagent (TaKaRa, Osaka, Japan), with U6 as an endogenous reference.

### 2.8. Silver Carp Head Kidney Macrophage Cell (HKC) Infection with LPS via miR-30b-5p

Silver carp head kidney macrophage cells (HKCs) were isolated and purified as previously described [29,30]. Briefly, three healthy silver carp were anesthetized with MS-222 (100 mg/L, Sigma-Aldrich), and the head kidney tissues were collected aseptically and washed repeatedly in precooled PBS. After that, the tissues were cut into pieces and rinsed through 100 μm sterile nylon mesh with L-15 medium (cytiva) containing 1% penicillin-streptomycin solution (100×, Beyotime, Nantong, China) and 2% fetal bovine serum (FBS). The cell suspension was loaded onto 34%/51% Percoll (Pharmacia, Santa Clara, CA, USA) density gradient and centrifuged at 500× *g* for 30 min. Thereafter, the cells were re-suspended in L-15 medium and centrifuged at 200× *g* for 10 min. Cell count was measured using hematometry, and activity was measured using trypan blue staining. Finally, the cells were inoculated in 6-well plates at a density of 4 × 10^7^ per well, and cultured in L-15 medium containing 1% penicillin–streptomycin solution (100×, Beyotime) and 2% FBS at 25 °C. After 12 h, L-15 complete medium containing 20% FBS was replaced for follow-up experiment.

The synthetic miR-30b-5p (100 nM, GenePharma, Shanghai, China) mimics or control mimics and inhibitor or control inhibitor were transfected into HKC. After 48 h of transfection, LPS (30 μg/mL, Sigma, St. Louis, MO, USA) was used to activate the cells and was stimulated for 6 h. With untreated cells serving as the CK, three biological replicates were used in each experiment. Afterward, total RNA was collected from the HKC, and the amounts of inflammatory cytokines (TNFα, IL-6, and IL-1β) expressed were monitored via relative qRT-PCR and standardized to the expression of GAPDH. The primer information is shown in Table S1.

### 2.9. Examining the Statistics

Every experiment was conducted independently at least three times (*n* ≥ 3). The 2^−ΔΔCt^ method was used to gauge the expression levels of miRNA and inflammatory factor. GraphPad Prism 9 software was used to analyze the data via unpaired two-tailed Student’s *t*-tests [31]. The results are presented as mean ± standard error (standard error of the mean, SEM), and a *p*-value of less than 0.05 indicates statistical significance in the difference between the two groups.

## 3. Results

### 3.1. High-Throughput Sequencing Results and miRNA Identification

**Figure 1:**
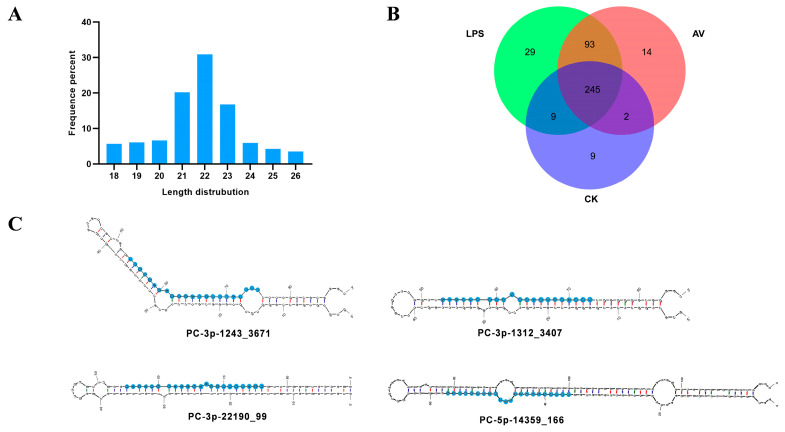
Characterization of miRNAs and the secondary structures of the novel predicted miRNAs. (**A**) Length distribution of all expressed miRNAs in silver carp. (**B**) Venn diagram of miRNAs from AV, LPS, and CK groups. (**C**) Secondary structures of the four novel predicted miRNAs.

To explore the silver carp miRNA expression profiles after Gram-negative bacterial infection, three groups (AV, LPS, and CK groups, each with three biological replicates) of spleen tissues were used to create and sequence miRNA libraries. Figure S1 shows the results of miRNA-seq principal component analysis. Following mass filtration, each library obtained about 3–13 million clean reads (Table S2). Correspondingly, the length distribution analysis of clean reads demonstrated that the length of most miRNAs was 21–23 nucleotides, with the majority being 22 nt, and the length distribution and frequency percentages were consistent with the typical length range of miRNAs (Figure 1A).

The results of miRNA sequencing produced 401 miRNAs (Figure 1B), including 397 known and 4 novel predicted miRNAs. Among them, the most abundant miRNAs were miR-143 (mean total reads of 714,579) and miR-21 (mean total reads of 656,554). Additionally, the secondary structures of the novel predicted miRNAs (PC-3p-1312_3407, PC-3p-1243_3671, PC-5p-14359_166, and PC-3p-22190_99) are shown in Figure 1C. However, their read counts are at a relatively low level.

### 3.2. Differentially Expressed miRNAs (DEmiRNAs)

**Figure 2:**
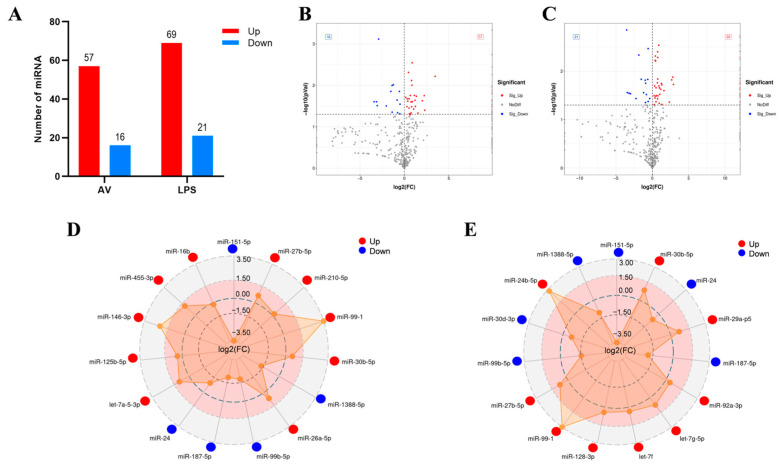
Expression patterns of the DEmiRNAs induced by challenge groups (AV and LPS). (**A**) Number of DEmiRNAs in different groups. “Up” and “Down” indicate up- and down-regulated expression, respectively. (**B**,**C**) Volcano plot of DEmiRNAs: CK vs. AV (**B**); CK vs. LPS (**C**); (**D**) the top-15 significant DEmiRNAs in AV group; (**E**) the top-15 significant DEmiRNAs in LPS group. Red spot: significant up-regulated expression (Sig-Up); blue spot: significant down-regulated expression (Sig-Down); and gray spot: no significant difference in expression (No Diff).

Three groups of DEmiRNAs were analyzed. In contrast to the CK group (*p* < 0.05), 57 and 16 DEmiRNAs were up- and down-regulated in the AV group, respectively, while 69 and 21 DEmiRNAs were up- and down-regulated in the LPS group, respectively (Figure 2A–C). Figure S2 shows the clustering results of DEmiRNAs. The top 15 miRNAs with appreciable variations in the infected groups (AV and LPS) were analyzed as shown in Figure 2D,E. Eight miRNAs with the same regulatory trend were screened (three up- and five down-regulated). Among them, the same down-regulated miRNAs were miR-151-5p, miR-24, miR-187-5p, miR-99b-5p, and miR-1388-5p, while the same up-regulated miRNAs were miR-30b-5p, miR-99-1, and miR-27b-5p (Table S3).

### 3.3. Identification and Functional Annotation of DEM Target Genes

**Figure 3:**
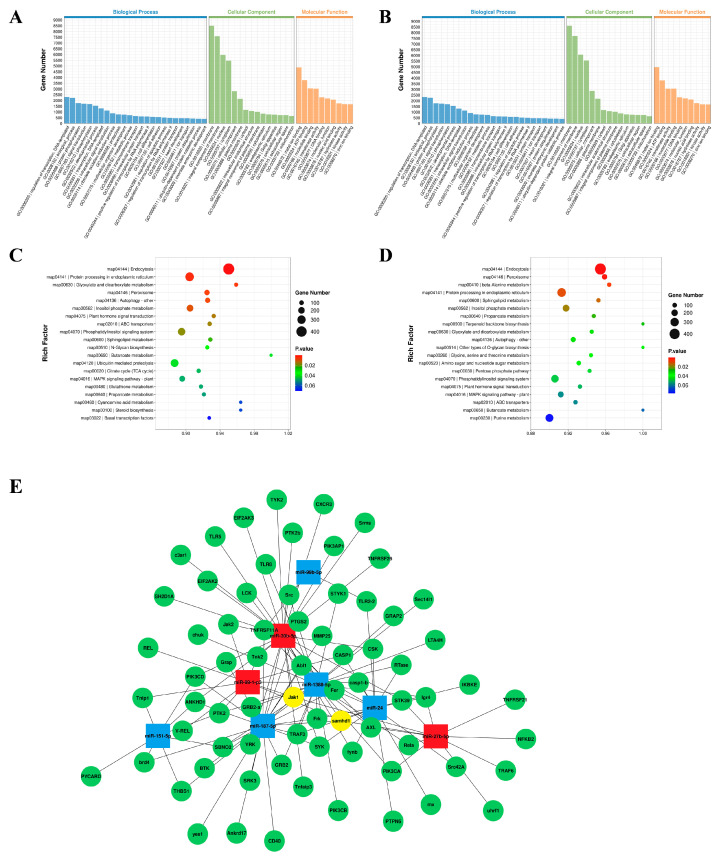
GO and KEEG enrichment analysis of DEmiRNA-predicted target mRNAs. (**A**,**B**) GO enrichment analysis of two challenge groups: CK vs. AV (**A**); CK vs. LPS (**B**); (**C**,**D**) KEEG enrichment analysis of two challenge groups: CK vs. AV (**C**); CK vs. LPS (**D**). Gene number: number of target genes in each pathway. Rich factor: the ratio of target genes to total genes in each pathway. (**E**) A network of miRNA–mRNA associated with the immune system. The squares indicate miRNAs; the red squares are up-regulated miRNAs and the blue squares are down-regulated miRNAs; the green circles are the target mRNAs for prediction and the yellow circles are the target genes for predicting miRNA co-regulation.

To understand the function of DEmiRNAs in silver carp, the target genes of DEmiRNAs with significant differences were predicted using TargetScan (v5.0) and miRanda (v3.3a). In this investigation, 51,392 genes were predicted to be DEmiRNAs’ potential targets. Afterwards, GO and KEGG enrichment analyses were carried out on the projected target genes to identify physiological processes that may be regulated by these DEmiRNAs. GO analysis showed that the DEmiRNAs’ target genes in the challenge groups (AV and LPS) had similar enrichment results, mainly in the membrane and integral components of the membrane (Figure 3A,B). An analysis of the top-20 KEGG pathway rankings has shown that the majority of the DEM targets were significantly enriched in endocytosis, protein processing in the endoplasmic reticulum and peroxisomes, etc. (Figure 3C,D). Additionally, some signaling pathways associated with innate immunity and inflammation were obtained, which included MAPK, the Jak/STAT signaling pathway, the autophagy signaling pathway, etc. Furthermore, numerous immune-related genes were identified, such as JAK1, PIK3, Toll-like receptors (TLR 5, TLR13), and NF-κB1. Finally, to assess the potential role of DEmiRNAs in the regulation of the immune response, we constructed an miRNA–mRNA interaction network focusing on the target genes associated with innate immunity (Figure 3E).

### 3.4. qRT-PCR Verification of DEmiRNAs

**Figure 4:**
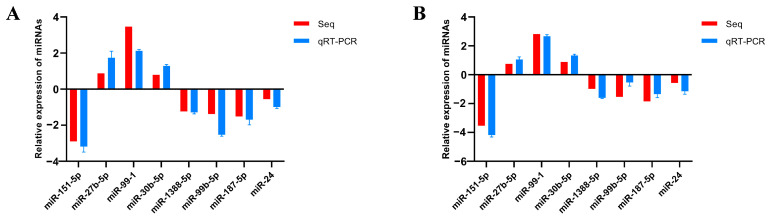
Differentially expressed miRNAs validated by qRT-PCR. (**A**) Validation of differentially expressed miRNAs (CK vs. AV); (**B**) validation of differentially expressed miRNAs (CK vs. LPS).

To verify the reliability of the sequencing results, eight DEmiRNAs were screened using U6 as an internal reference. The expression level of DEmiRNAs was detected via qRT-PCR; thereafter, the outcomes were contrasted with those of high-throughput sequencing analysis. The results confirmed that the expression profiles of these DEmiRNAs were significantly similar to those obtained using high-throughput sequencing analysis (Figure 4). There are some differences between these two methods. However, the trends in change are essentially the same. Overall, the qRT-PCR results confirm the RNA-seq results and support the reliability of the DEmiRNAs.

### 3.5. Effect of miR-30b-5p on Inflammation Induction

**Figure 5:**
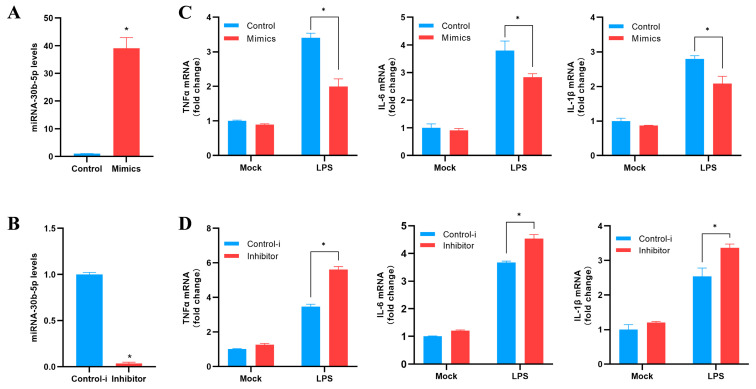
miR-30b-5p negatively regulates the expression of inflammatory cytokines. (**A**,**B**) Transfection of silver carp HKC with control mimics (control) and miR-30b-5p mimics (mimics) (**A**) or control inhibitor (control-i) and miR-30b-5p inhibitor (inhibitor) (**B**) at a final concentration of 100 nM. After 48 h, qRT-PCR was used to measure the expression of miR-30b-5p and normalize it to U6. (**C**,**D**) Mimics or control (**C**) and inhibitor or control-i (**D**) were transfected into HKC for 48 h. Following LPS stimulation of HKC, qRT-PCR was used to determine the mRNA expression levels of TNF-a, IL-6, and IL-1β, which were then normalized by GAPDH expression. In control cells, results were standardized to 1. The average and standard errors of three separate triplicate experiments are used to represent all data. Compared to controls, * *p* < 0.05.

Based on previous experimental results, we chose miR-30b-5p to investigate its function in the host antibacterial immune response. We evaluated the effect of miR-30b-5p overexpression or inhibition on inflammatory cytokines in silver carp HKC stimulated via LPS. Firstly, the effects of the synthesized exogenous miR-30b-5p mimics and inhibitor on the expression of endogenous miR-30b-5p in HKC were evaluated. Compared to the CK, the mimics significantly increased the expression of miR-30b-5p in silver carp HKC (Figure 5A), while the inhibitor significantly decreased the endogenous expression (Figure 5B). Afterwards, we investigated the impact of the miR-30b-5p mimics or inhibitor on the amounts of inflammatory cytokines expressed in LPS-stimulated HKCs. The findings suggested that the transfection of miR-30b-5p mimics reduced the production of LPS-induced TNFα, IL-6, and IL-1β (Figure 5C). In comparison, the miR-30b-5p inhibitor significantly up-regulated the expression levels of inflammatory cytokines (Figure 5D). According to the data presented, miR-30b-5p functions as a negative regulator that controls the release of inflammatory cytokines by LPS-stimulated HKCs. These results suggest that miR-30b-5p may inhibit excessive immune responses by inhibiting the expression of inflammatory cytokines and protecting the body.

## 4. Discussion

Infection with pathogenic bacteria is one of the key factors limiting the development of teleost fish culture. MiRNAs play an important role in regulating the immune response. Numerous studies have established that miRNAs participate in the immune responses of many teleost fish caused by pathogenic bacteria infection [12,22]. However, there is no related report in silver carp. In addition, most of the common pathogens in aquaculture are Gram-negative bacteria. The ubiquitous Gram-negative bacterial pathogen *A. veronii*, which can infect many fish species, results in high mortality and significant economic losses [5,6,7,9]. Therefore, this study identified the expression profiles of miRNA in silver carp stimulated by *A. veronii* and LPS, respectively, by screening miRNAs that may participate in the immune responses of silver carp. This study explores miRNA regulation in silver carp during pathogenic bacterial infection and provides a reference for the future development of non-coding RNA antibacterial drugs.

MiRNA is evolutionarily conserved across species, with increasing evidence showing that they can be promising phylogenetic markers [32]. In this study, 397 known miRNAs were identified via sequencing, of which the miRNA-143/145 cluster and miRNA-21 belonged to two conserved vertebrate miRNA families and were abundantly expressed in each group. Previous studies have shown that the miR-143/miR-145 cluster is widely implicated in cancer regulation [33]. MiR-143 may also regulate autoimmune diseases and the inflammatory response by interacting with target genes [34]. MiR-21 in teleost fish has been extensively investigated, and miR-21 can target toll-like receptors (TLR) TLR28 [35] and IRAK4 [36] to regulate the inflammatory response. Consequentially, these abundant miRNAs might be crucial to the silver carp’s defense against infection. In addition, four novel miRNAs were screened, and the secondary structure was predicted. It is worth noting that these novel miRNAs have low expression. Previous research has indicated that a low abundance of miRNA may be associated with the spatial, temporal, physiological, and histological regulation of miRNA expression [37]. Therefore, the novel miRNAs in silver carp identified in this research can have a significant impact on other types of functional regulation, which need to be studied in the future.

Innate immunity is the first barrier in all multicellular organisms that resists exogenous infections. Many studies have indicated that microRNAs play an important regulatory role in innate immunity and the inflammatory response to pathogen infection in teleost fish. By comparing the top-15 DEmiRNAs in the infected groups (LPS and AV), 8 identical miRNAs were screened (3 up-regulated and 5 down-regulated) (Figure 2D,E). A number of these miRNAs have been demonstrated to have a tight connection to immunomodulation. The miR-99 family (miR-99a, miR-99b, and miR-100) has been shown to play a regulatory role in cell invasion and migration [38]. For defense against infections, cell migration and activation are essential. Alan et al. have found that miR-99 reduces the chemotaxis of zebrafish neutrophils and human neutrophil-like cells and increases the susceptibility of zebrafish to bacterial infections [39]. Furthermore, a down-regulated miR-24 expression has been observed in PM2.5-induced zebrafish (Danio rerio) [40]. It has been shown that miR-1388 is involved in the immune regulation of yellow catfish (*Pelteobagrus fulvidraco*) challenged with *Edwardsiella tarda* infection and that its expression level is down-regulated [41]. Chang et al. have demonstrated that microRNA-1388-5p inhibits the NF-κ B signaling pathway of miiuy croaker by targeting IRAK1, and miR-1388 is up-regulated at different time points of LPS stimulation [42]. MiRNA has evolutionary conservatism. However, different fish have different miRNA effects in response to bacterial infection, and the same fish may have different miRNA responses to different bacterial pathogens, which vary with different stimulant concentrations and infection times, etc. In this study, miRNA-1388-5p was down-regulated under the stimulation of AV and LPS; the specific mechanism of action needs to be further investigated in future research. The miR-30 family regulates autophagy, apoptosis, oxidative stress, and inflammation in mammals [43,44,45]. With the advancement of research, immunomodulation of the miR-30 family in teleost fish has been widely verified. The overexpression of miR-30b in *Epinephelus coioides* promotes nervous necrosis virus (NNV) replication [46]. Changes in the immune response of the miR-30 family during environmental stress and development have been recorded. Alice et al. proposed that the miRNA-30 family is an important post-transcriptional regulator of parr–smolt transformation and seawater adaptation, and their enrichment analysis of target mRNAs showed that several biological processes are associated with the immune system [47]. In this work, it was speculated that miR-30b-5p’s high expression had a regulatory function in the silver carp’s antibacterial response.

The regulatory functions of miRNAs are mainly mediated by target genes. According to GO and KEGG enrichment studies of their target mRNAs, the majority of these differentially expressed target mRNAs are prevalent in a variety of biological activities involving lipid metabolism and immune function. The majority of miRNA targets are much more abundant in immune-related signaling pathways such as endocytosis, protein processing in the endoplasmic reticulum, and peroxisomes. Relevant studies have shown that metabolism-related signaling cascades, including glycolysis, oxidative phosphorylation, the tricarboxylic acid cycle, amino acid metabolism, and lipid metabolism, are interacting pathways that are vital in inducing innate immune responses and creating immunological memory [48]. Here, an analysis of KEGG pathways has revealed that numerous miRNAs are abundant in metabolic pathways. In addition, a number of signaling pathways associated with the innate immune and inflammatory responses have been identified, including the MAPK pathway, the autophagy pathway, and the Jak/STAT pathway. Finally, to assess the potential function of DEmiRNAs in immune response regulations, we focused on target genes connected with the inflammatory and innate immune responses and we constructed miRNA–mRNA interaction networks. Previous studies have shown that numerous miRNAs can target a single mRNA, while a single miRNA can target multiple mRNAs. Many types of miRNAs are involved in the immune regulation mechanism following pathogen infection, and the synergistic regulation among various miRNAs is of great importance in studying the mechanism of post-transcriptional regulation of miRNAs induced by infections [49]. MiRNA–mRNA interaction network analysis has revealed that several miRNAs share the same target genes, e.g., JAK1 (miR-27b-5p, miR-99-1, miR-30b-5p, miR-1388-5p, and miR-187-5p) and SAMHD1 (miR-27b-5p, miR-99-1, miR-1388-5p, miR-187-5p, miR-24, and miR-1388-5p). The composition and characteristics of fish JAKs are comparable to those of mammals. As a key component of the JAK/STAT pathway, fish JAKs can be induced by viruses and bacteria and play an important role in the inflammatory and antiviral responses. It has been reported that JAK1 is activated in *Megalobrama amblycephala* following *Aeromonas hydrophila* infection. It is speculated that the JAK1/STAT pathway mediated by IL-6R may regulate the immune response [50]. SAMHD1 is an innate immune-limiting factor and mediates antiviral and apoptotic responses via IRF3. Yang et al. have shown that SAMHD1 interacts with the IKK complex to inhibit NF-κ B from being activated during the inflammatory response and viral infection [51]. SAMHD1 has also been shown to interact with VDAC1 to inhibit acute inflammation induced by LPS [52]. However, additional study is required to verify the miRNA–mRNA interaction.

The inflammatory response is one of the major responses induced by pathogenic bacterial infections. The host cells initiate intracellular signal responses via pattern-recognition receptors (PRRs) and ultimately produce inflammatory factors to resist pathogen infection [53]. As models for in vitro study, cell lines have been utilized to research the virology, pathology, immunology, and developmental biology of vertebrates [8]. Previous reports demonstrate that fish head kidney macrophage cells can express immune-related genes and have significant functions in the regulation of innate and adaptive immunity [29,54]. In recent years, head kidney macrophages have been selected as model cells to study the regulation mechanism of miRNA in a variety of teleosts [12,35,36], whereas there is no published information on the mechanism of miRNA infection with pathogenic bacteria in silver carp. This study has revealed that miR-30b-5p is a negative regulator involved in the inflammatory response, and that it negatively regulates the expression of pro-inflammatory cytokines (TNFα, IL-6, and IL-1β) in HKCs. Previous research has shown that an appropriate immune and inflammatory response is an effective means of eradicating pathogenic bacterial infections, but excessive inflammation can lead to immune dysregulation. Thus, various negative regulators, or paracrine mechanisms, are required to maintain immune system homeostasis. Consistent with the results of this study, the negative feedback regulatory mechanism of miRNAs was demonstrated by miRNA-144 [55] in Japanese flounder, and miR-217 [56] and miR-2187 [57] in *Miichthys miiuy*. We determined that most of the DEmiRNAs in the Gram-negative bacterial infection model constructed in this study were up-regulated in spleen expression, including the screened miR-30b-5p (*p* < 0.01). It is hypothesized that the up-regulation of miR-30b-5p may be due to the body’s self-protection against excessive inflammation, which results in negative feedback regulation of the antimicrobial response in silver carp. Reduced inflammation brought on by pathogen infection depends on this endogenous negative regulatory mechanism. It not only enriches the miRNA–mRNA regulation network in teleost fish but also provides a new idea to study the pathogen regulatory mechanism mediated by miRNAs. In addition, it has been reported that miRNA-30b-5p regulates the immune response mainly through its targeting of immune-related genes, e.g., extracellular vesicle-associated microRNA-30b-5p regulates the inflammatory responses of macrophages through the SIRT1/NF-κ B pathway [58]. By directly targeting NLRP3, miR-30b-5p participates in the CB1-mediated activation of the macrophage NLRP3 inflammasome [59]. Hence, we predicted the target genes related to the immunity of miR-30b-5p in this study (Figure 3E), and the regulating mechanism remains to be further studied.

Currently, most studies involving miRNA screening and identification are performed using high-throughput sequencing technology. Sequencing can directly reflect the abundance of miRNAs, and the reliability of the results has been clinically validated with a large amount of data. Databases on the statistical analysis of miRNAs in teleost fish are still incomplete compared to mammalian ones. Therefore, this requires continuous research and enrichment. Teleost miRNAs have been shown to have a similar mechanism of action to mammalian miRNAs. A single miRNA can directly or indirectly affect the protein synthesis of multiple genes, while multiple miRNAs can act together on multiple signaling pathways of a single gene protein to perform various regulatory functions [53]. Therefore, the research focus of our future work will be the in-depth examination of some miRNAs that can regulate numerous target genes and multiple signaling pathways simultaneously. Moreover, with the deepening of miRNA research and a variety of ncRNAs (lncRNA and circRNA), the role of ceRNA mechanisms such as the teleost species’ lncRNA–miRNA–mRNA axis has also received considerable attention. It has been reported to be important in the regulation of growth and development, skeletal muscle, reproduction, pathogen infection, and other factors related to stress immunological responses [60]. The mechanism of teleost ncRNA (including mirna, lncrna, and circrna) network regulation will be emphasized in future aquaculture research.

## 5. Conclusions

In this research, we provided the first-ever characterization of DEmiRNAs in silver carp during Gram-negative bacterial infection by comparing two infection-stimulated groups (LPS and AV). MiR-30b-5p, one of the eight candidate DEmiRNAs, which may play a broad regulatory role in Gram-negative bacterial infections, was demonstrated to negatively regulate the LPS-induced inflammatory response in HKCs. These results lay the foundation for further study of the silver carp microRNA regulatory mechanism during pathogenic bacterial infection and provided a reference for the future development of non-coding RNA antibacterial drugs.

## Data Availability

Data will be supplied upon request.

## Supplementary Materials

The following supporting information can be downloaded at https://www.mdpi.com/article/10.3390/ani14020285/s1, Figure S1: Principal Component Analysis (PCA) of miRNA-seq results. Figure S2: The clustering figures of DEmiRNAs in two challenge groups. (A) CK vs. AV; (B) CK vs. LPS. Red indicates high expression of miRNAs, dark blue indicates low expression of miRNAs. Table S1: qRT-PCR primer sequence information in this study. Table S2: The library information in this study. Table S3: The top-15 significant DEmiRNAs in the group (AV vs. CK). Table S4: The top-15 significant DEmiRNAs in the group (LPS vs. CK).
