# Supplementary material for: Identification and Characterization of Immune-Associated MicroRNAs in Silver Carp (Hypophthalmichthys molitrix) Responding to Aeromonas veronii and LPS Stimulation
Source: Animals (Basel). 2024 Jan 17;14(2):285. doi: 10.3390/ani14020285 (PMC10812751; doi:10.3390/ani14020285)
Supplement: Supplementary file 1 [file animals-14-00285-s001.zip › animals-2747312-supplementary.pdf]

Appendix A. Supplementary Materials

Primer ID	Sequences (5' to 3')

Identification and Characterization of Immune-associated miRNAs in Silver Carp (*Hypophthalmichthys molitrix*) Responding to *Aeromonas veronii* and LPS Stimulation

Meng Liu[†], Huan Tang[†], Kun Gao, Xiqing Zhang, Zihan Yang, Xiaofeng Shan[*] and Yunhang Gao[*]

College of Veterinary Medicine, Jilin Agricultural University, Changchun 130118, China. liumeng4610@163.com (M.L.), tanghuan202308@163.com (H.T.), KunGao213@163.com (K.G.), Zhangxiqing1020@163.com (X.Z.), yyangzihan622@163.com (Z.Y.)

* Corresponding authors: *E-mail*: gaoyunhang@163.com (Y. G.), sxf1997@163.com (X.S.).

Author to whom correspondence should be addressed.gaoyunhang@163.com (Y.G.), Tel.: +86 13159752912 (Y.G.).

[†] Each of these authors has contributed equally to this work.

Table S1. qRT-PCR primer sequence information in this study

RT-miR-151-5p	GTCGTATCCAGTGCAGGGTCCGAGGTATTCGCACTGGATACGACACTAGA
miR-151-5p-F	CGCGTCGAGGAGCTCACAG
RT-miR-27b-5p	GTCGTATCCAGTGCAGGGTCCGAGGTATTCGCACTGGATACGACGTTCAC
miR-27b-5p-F	CGCGAGAGCTTAGCTGATTG
RT-miR-99-1-p3	GTCGTATCCAGTGCAGGGTCCGAGGTATTCGCACTGGATACGACGAGACC
miR-99-1-p3-F	CGCGCAAGCTCGTTTCTATG
RT-miR-30b-5p	GTCGTATCCAGTGCAGGGTCCGAGGTATTCGCACTGGATACGACAGCTGA
miR-30b-5p-F	GCGCGTGTAAACATCCTACAC
RT-miR-1388-5p	GTCGTATCCAGTGCAGGGTCCGAGGTATTCGCACTGGATACGACACATTC
miR-1388-5p-F	GCGAGGACTGTCCAACCTGA
RT-miR-99b-5p	GTCGTATCCAGTGCAGGGTCCGAGGTATTCGCACTGGATACGACGCAAGT
miR-99b-5p-F	GCGAACCCGTAGATCCGA
RT-miR-187-5p	GTCGTATCCAGTGCAGGGTCCGAGGTATTCGCACTGGATACGACCCATGT
miR-187-5p-F	GCGGGCTGCAACACAGG
RT-miR-24	GTCGTATCCAGTGCAGGGTCCGAGGTATTCGCACTGGATACGACACTGTT
miR-24-F	CGTGGCTCAGTTCAGCAGG
Universal primer-R	AGTGCAGGGTCCGAGGTATT
U6-F	CTCGCTTCGGCAGCACA
U6-R	AACGCTTCACGAATTTGCGT
TNF-α-F	CAGGGCTGCCATCCATTTA
TNF-α-R	AAGCCGCCTGAAGAGAAAG
IL6-F	CGTATGAAGGTGTCAGGATCAG
IL6-R	CCACGTCAGGACACTGTAAAT
IL1β-F	GGTGAAGAAGAGGTGCTCAA
IL1β-R	AAGGTCGTGCTGGTCTTATC
GAPDH-F	GGGATTGTCGCTCATCTATCTT
GAPDH-R	GTTGCTGTACCCAAACTCATTG

Table S2. The library information in this sudy

sample	Raw reads	3ADT&length filter	Junk reads	Clean reads	Rfam	mRNA	Repeats	valid reads
CK1	13819794	7438749	67466	6313579	542111	1322382	1227	4719375
CK2	9662066	5707222	118355	3836489	768205	1887821	1233	1426761
CK3	16977352	11828177	75149	5074026	631364	1738721	1338	2991109
AV1	19244994	12105695	8736	7130563	328224	2793513	3412	4064183
AV2	11177077	5763713	36258	5377106	429047	1501152	1193	3690472
AV3	19848401	6151425	19797	13677179	626108	4405500	6642	8913754
LPS1	15820469	10035795	22596	5762078	584508	2430017	2715	3104373
LPS2	15986532	7376376	24105	8586051	728380	3033965	3105	5221597
LPS3	20915196	7047560	23939	13843697	1056832	4991017	5106	8271890

3ADT&length filter: reads removed due to 3ADT not found and length with <18 nt and >25 nt were removed (for plants); length with<18 and >26nt were removed (for animals).

Junk reads: Junk: >=2N, >=7A, >=8C, >=6G, >=7T, >=10Dimer, >=6Trimer, or >=5Tetramer.

Clean reads: equal to raw reads - 3ADT&length filter - Junk reads.

Rfam: Collection of many common non-coding RNA families except microRNA; http://rfam.janelia.org.

Repeats: Prototypic sequences representing repetitive DNA from different eukaryotic species; http://www.girinst.org/repbase.

Table S3. The top 15 significant DEmiRNAs in the group (AV vs. CK)

miR_name	miR_seq	up/down	fold_change(AV(mean)/CK(mean))	log2(fold_change)	pvalue(t_test)	CK(mean)	AV(mean)
**miR-151-5p**	TCGAGGAGCTCACAGTCTAGT	down	0.13	-2.89	0.0008	44	6
**miR-27b-5p**	AGAGCTTAGCTGATTGGTGAAC	up	1.84	0.88	0.0029	183	337
miR-210-5p	AGCCACTGACTAACGCACATTG	up	1.38	0.47	0.0049	250	346
**miR-99-1**	CAAGCTCGTTTCTATGGGTCTC	up	11.06	3.47	0.0061	8	86
**miR-30b-5p**	TGTAAACATCCTACACTCAGCT	up	1.74	0.80	0.0077	11,261	19,628
**miR-1388-5p**	AGGACTGTCCAACCTGAGAATGT	down	0.43	-1.23	0.0097	735	312
miR-26a-5p	TTCAAGTAATCCAGGATAGGCTTTT	up	1.82	0.86	0.0099	243	441
**miR-99b-5p**	AACCCGTAGATCCGAACTTGC	down	0.38	-1.38	0.0101	164	63
**miR-187-5p**	GGCTGCAACACAGGACATGG	down	0.35	-1.52	0.0140	804	281
**miR-24**	TGGCTCAGTTCAGCAGGAACAGT	down	0.68	-0.56	0.0140	2,659	1,802
let-7a-5-3p	CTATACAGTCTATTGCCTTCCT	up	2.18	1.12	0.0176	142	309
miR-125b-5p	TCCCTGAGACCCTAACTTGTGA	up	1.59	0.66	0.0176	15,359	24,346
miR-146-3p	ATCTATGGGCTCAGTTCTTCTG	up	4.72	2.24	0.0177	111	523
miR-455-3p	TGCAGTCCATGGGCATATACAC	up	2.56	1.35	0.0184	72	184
miR-16b	TAGCAGCATGTAAATATTGGA	up	1.11	0.15	0.0196	6,092	6,771

red is p value<0.001,orange is p value <0.01,blue is p value<0.05

Table S4. The top 15 significant DEmiRNAs in the group (LPS vs. CK)

miR_name	miR_seq	up/down	fold_change(LPS(mean)/CK(mean))	log2(fold_change)	pvalue(t_test)	CK(mean)	LPS(mean)
**miR-151-5p**	TCGAGGAGCTCACAGTCTAGT	down	0.09	-3.53	0.0014	44	4
**miR-30b-5p**	TGTAAACATCCTACACTCAGCT	up	1.85	0.89	0.0029	11,261	20,862
**miR-24**	TGGCTCAGTTCAGCAGGAACAGT	down	0.67	-0.57	0.0034	2,659	1,786
miR-29a-p5	ACTGATTTCCTCTGGTGCTGAGA	up	1.67	0.74	0.0040	475	791
**miR-187-5p**	GGCTGCAACACAGGACATGG	down	0.28	-1.85	0.0047	804	223
miR-92a-3p	TATTGCACTTGTCCCGGCCTGT	up	1.34	0.42	0.0049	22,888	30,648
let-7g-5p	TGAGGTAGTAGTTTGTATAGTTA	up	1.68	0.74	0.0052	487	815
let-7f	TGAGGTAGTAGTTTGTATAGTT	up	1.31	0.39	0.0060	25,049	32,820
miR-128-3p	TCACAGTGAACCGGTCTCTTT	up	1.37	0.46	0.0062	5,574	7,652
**miR-99-1**	CAAGCTCGTTTCTATGGGTCTC	up	7.06	2.82	0.0133	8	55
miR-27b-5p	AGAGCTTAGCTGATTGGTGAAC	up	1.68	0.75	0.0146	183	307
**miR-99b-5p**	AACCCGTAGATCCGAACTTGC	down	0.35	-1.53	0.0147	164	57
**miR-30d-3p**	CTTTCAGTCGGATGTTTGCAGC	down	0.65	-0.63	0.0148	4,195	2,713
miR-24b-5p	TGCCTGCTGAACTGGTATCAGT	up	6.34	2.66	0.0151	14	89
**miR-1388-5p**	AGGACTGTCCAACCTGAGAATGT	down	0.51	-0.98	0.0154	735	373

red is p value<0.001,orange is p value <0.01,blue is p value<0.05.

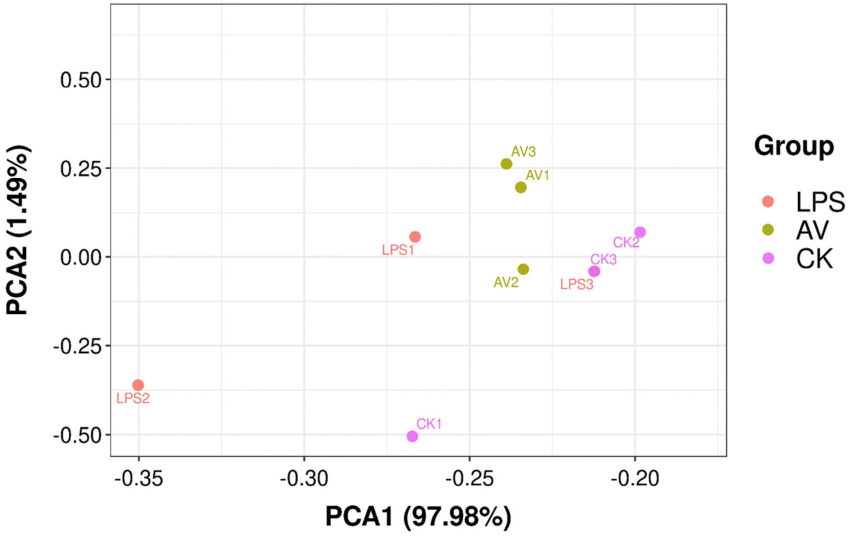

Figure S1. Principal Components Analysis (PCA) of miRNA-seq results.

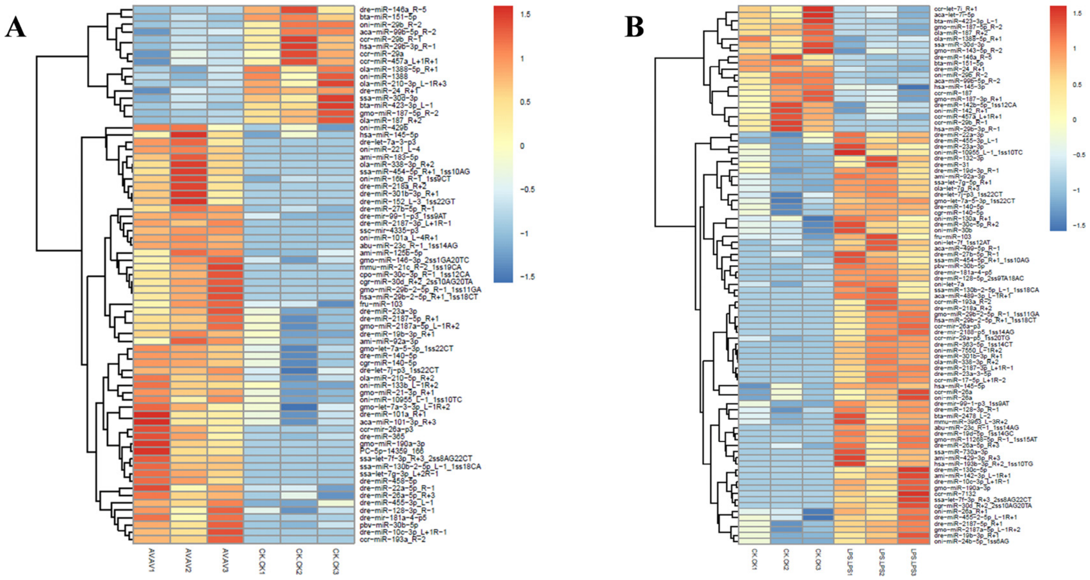

Figure S2. The clustering figures of DEmiRNAs in two challenge groups. (A) CK vs. AV; (B) CK vs. LPS; Red indicates high expression of miRNAs, dark blue indicates low expression of miRNAs.
